# Impact of preoperative MRI on long-term outcomes of breast-conserving therapy with electronic kV-intraoperative radiotherapy: A risk-stratified analysis based on ASTRO criteria

**DOI:** 10.1016/j.breast.2026.104840

**Published:** 2026-06-15

**Authors:** Hsin-Yi Yang, Yi-Li Lin, Cheng-Yen Lee, Li-Chung Yang, Yu-Chen Hsu

**Affiliations:** a Clinical Data Center, Ditmanson Medical Foundation Chia-Yi Christian Hospital, No.539, Zhongxiao Rd., East Dist., Chiayi City, 60002, Taiwan; b Department of Radiology, Ditmanson Medical Foundation Chia-Yi Christian Hospital, No.539, Zhongxiao Rd., East Dist., Chiayi City, 60002, Taiwan; c Department of Radiation Oncology, Ditmanson Medical Foundation Chia-Yi Christian Hospital, No.539, Zhongxiao Rd., East Dist., Chiayi City, 60002, Taiwan; d Department of Surgery, Ditmanson Medical Foundation Chia-Yi Christian Hospital, No.539, Zhongxiao Rd., East Dist., Chiayi City, 60002, Taiwan

**Keywords:** Breast-conserving surgery, Pre-operative MRI, Recurrence, ASTRO-Risk stratification, Radiation therapy

## Abstract

**Purpose:**

Preoperative breast MRI (pMRI) in patients undergoing breast-conserving surgery (BCS) remains controversial, particularly regarding long-term oncologic outcomes. The utility of pMRI within contemporary, risk-stratified radiation therapy paradigms, using the ASTRO risk classification as a reference, is unclear.

**Methods and materials:**

We retrospectively analyzed 613 BCS patients at a single institution (2014–2022). Patients were stratified by pMRI status and ASTRO 2016 risk classification based on final pathology. Primary outcomes included overall recurrence, locoregional recurrence, distant metastasis, and overall survival. Cox proportional hazards regression estimated hazard ratios (HRs) and 95% confidence intervals (CIs).

**Results:**

Overall recurrence was lower in the pMRI cohort versus non-pMRI (4.0% vs. 11.2%, p = 0.007). Among high-risk patients, pMRI was associated with reduced overall recurrence (5.3% vs. 14.6%, p = 0.009) and a trend toward lower locoregional recurrence (3.8% vs. 9.4%, p = 0.066). This benefit was observed in high-risk patients receiving standard radiotherapy (4.9% vs. 13.1%, p = 0.024) but not in those receiving non-standard radiotherapy. No significant benefit was seen in low-risk patients. Adjusted analysis identified pMRI as an independent protective factor for recurrence (HR 0.42, 95% CI 0.19–0.93, p = 0.036).

**Conclusions:**

pMRI significantly reduces recurrence in high-risk BCS patients, particularly with standard radiotherapy. These findings support using ASTRO risk classification to guide selective pMRI use, prioritizing high-risk patients where precise surgical planning and target delineation yield the greatest clinical benefit.

## Highlights

• Preoperative MRI reduced recurrence in ASTRO high-risk patients.
• No significant oncologic benefit was observed in low-risk patients.
• pMRI benefit disappeared when high-risk patients received non-standard RT.
• pMRI optimized local control but could not replace adequate radiotherapy.
• ASTRO risk stratification may guide selective pMRI utilization.

## 1 Introduction

Breast-conserving surgery (BCS) followed by adjuvant radiation therapy (RT) remains the standard of care for the majority of early-stage breast cancer patients, achieving equivalent survival rates to mastectomy while preserving quality of life [1]. Optimal local control requires a clean post-surgical residual breast [2] and thorough elimination of residual microscopic disease by RT [3]. Consequently, accurate pre-operative assessment is paramount [4]. While conventional mammography and ultrasound are standard, the use of pre-operative breast magnetic resonance imaging (pMRI) has significantly increased due to its high sensitivity for detecting occult disease and assessing the full extent of the tumor burden [5,6]. Despite its high sensitivity, the routine application of pMRI remains highly debated due to uncertain long-term outcome benefits [7,8].

The long-term oncologic benefit of pMRI is inconsistent across patient populations, reflecting different residual breast risk based on intrinsic tumor biology and clinical factors [4,9,10]. Some studies suggest that in high-risk patients, pMRI could detect occult lesions in residual breast tissue [4,11], potentially reducing residual occult disease following lumpectomy [9,12]. Conversely, the high inherent risk of residual or occult disease in the high-risk group means that failure to detect such lesions may lead to a higher rate of treatment failure [13].

On the other hand, the clinical utility of pMRI is also critical in low-risk patients considering de-escalated adjuvant RT. Although pMRI is not required in the American Society for Radiation Oncology (ASTRO) Accelerated Partial Breast Irradiation (APBI) guideline [14], some current APBI guidelines recommend pMRI screening to confirm patient suitability [[15], [16], [17]], specifically to evaluate if standard RT can be safely omitted. This reflects the inherent risk: de-escalated RT could fail in residual breast tissue if it contains occult lesions only detectable by pMRI [3,18]. Therefore, pMRI plays a dual and important role: it acts as a screening tool to detect occult disease in high-risk patients and as an essential confirmation step for safe treatment de-escalation in low-risk patients.

The ASTRO risk classification provides a robust framework for stratifying patients, guiding the selection of RT coverage for residual breast tissue [14,18]. While ASTRO updated its APBI guidelines in 2024, the 2016 risk stratification was maintained for this study as it remains the foundational framework for our institutional kV-IORT protocol [14]. However, its utility as a reference framework for pMRI selection remains undefined [4,15], especially considering the clinical-pathological discordance often encountered postoperatively. Therefore, an analysis focusing on ASTRO-stratified cohorts is essential to accurately assess the long-term impact of pMRI based on the differential residual breast risk and define its true value across the spectrum of risk categories.

The purpose of this retrospective study was to analyze the long-term oncologic outcomes after BCS, specifically stratifying them according to the ASTRO risk criteria and pMRI utilization. By comparing pMRI utilization across different risk groups, we aim to provide an empirically supported framework that determines which patients benefit most from pre-operative imaging, ultimately offering clear guidance on the optimal integration of pMRI into the breast cancer treatment paradigm for improved resource allocation and treatment efficacy.

## 2 Methods

### 2.1 Study design and patient population

This retrospective cohort study was conducted at a single-center hospital in Taiwan, reviewing all patients who underwent breast-conserving surgery for invasive breast cancer between January 2014 and December 2022. This study was approved by the Institutional Review Board of the study hospital, with a waiver of informed consent due to the retrospective nature of the study (IRB number: 2023029). Inclusion criteria were: (1) histologically confirmed invasive breast carcinoma; (2) treatment with breast-conserving surgery; (3) complete medical records including tumor characteristics, treatment details, and follow-up data; and (4) minimum follow-up of 12 months or until recurrence/death. Exclusion criteria included: (1) refusal of adjuvant radiotherapy following BCS; (2) prior history of breast cancer; (3) bilateral breast cancer; (4) incomplete treatment or follow-up records.

### 2.2 MRI protocol and imaging evaluation

pMRI was performed at the discretion of the treating physician, typically based on clinical suspicion of multifocal disease, high breast density, or young age, and finalized through shared decision-making between the physician and the patient. For patients undergoing neoadjuvant systemic therapy (NST), the preoperative MRI was performed during the initial staging workup, prior to the commencement of any systemic treatment, to establish a baseline for tumor extent and to guide subsequent surgical planning. MRI studies were conducted using a 1.5T scanner with dedicated breast coils, following standardized protocols including T1-weighted, T2-weighted, and dynamic contrast-enhanced sequences. All images were interpreted by a single, highly experienced board-certified radiologist (Y.L. Lin) specializing in breast imaging, thereby ensuring high diagnostic consistency across the cohort. Background Parenchymal Enhancement (BPE) levels were documented to assess their potential impact on imaging interpretation.

### 2.3 Risk stratification

Patients were retrospectively stratified according to the ASTRO 2016 consensus guidelines based on final pathology for accelerated partial breast irradiation [9]. The “Suitable” group (low-risk) was defined as meeting all ASTRO suitable criteria: age ≥50 years, positive hormone status, tumor size ≤2 cm, node-negative disease, negative margins, and no lymphovascular invasion (LVI). Patients not meeting all suitable criteria were classified as high-risk, encompassing both the “Cautionary” and “Unsuitable” categories.

Within the high-risk category defined by the ASTRO criteria, patients in this study were further classified into two subgroups based on the clinical predictability of their risk status.

(1) The clinically suspicious high-risk group: patients with pre-existing clinical indicators of high-risk status, including young age (<50 years), tumor size >2 cm on imaging, suspicious lymph node metastasis, or triple-negative/HER2-positive status on core biopsy. This group also includes patients whose high-risk status was confirmed by discordant postoperative findings, such as unexpected pathological upstaging in tumor size or nodal involvement.
(2) The pathologically-driven high-risk group: patients whose high-risk status is exclusively determined by occult pathological features—specifically LVI—despite fulfilling all other favorable clinical indicators (e.g., age ≥50 years, hormone receptor positivity, tumor size ≤2 cm, and negative nodal/margin status).

### 2.4 Radiation therapy classification

Radiation therapy was categorized as either standard or non-standard based on its adequacy for specific risk groups, following the 2016 ASTRO consensus guideline recommendations. Standard radiation therapy included conventional whole-breast radiotherapy (WBRT) with a boost delivered via either external beam or IORT. For patients in the low-risk (suitable) category, APBI via standalone IORT was also considered standard RT. Conversely, for the high-risk group, IORT alone without subsequent WBRT was classified as non-standard RT.

In our institution, IORT was performed using the Xoft Axxent eBx delivery system, which delivered a dose of 20 Gy to the tumor cavity surface with a 50 kV X-ray beam energy. For patients with pathologically confirmed nodal involvement, regional nodal irradiation was routinely integrated into the RT plan. This classification reflects the adherence to evidence-based radiation practices and the specific technical application of electronic IORT during the study period.

### 2.5 Data collection and outcomes

Comprehensive data were extracted from electronic medical records, including patient demographics, tumor characteristics (size, hormone receptor status, HER2 status, lymph node involvement, LVI), surgical margin status, systemic therapy (chemotherapy, hormone therapy, targeted therapy), and radiation therapy parameters.

Oncological outcomes evaluated in this study included overall recurrence—comprising locoregional and distant failures—as well as locoregional recurrence involving the ipsilateral breast or regional lymph nodes. Secondary endpoints included the incidence of distant metastasis and overall survival. Follow-up data were obtained through scheduled clinic visits, imaging studies, and review of medical records. Patients were followed for a maximum of 5 years after surgery. Recurrence was confirmed histologically or radiologically according to standard clinical criteria.

### 2.6 Statistical analysis

Baseline characteristics were compared between pMRI and non-pMRI groups using chi-square tests for categorical variables and t-tests or Mann-Whitney U tests for continuous variables. Recurrence rates were compared using chi-square or Fisher's exact tests as appropriate. Patients were stratified into four groups based on MRI status (yes/no) and risk classification (high-risk/low-risk). Outcomes were compared across these groups overall and within RT subgroups. Cox proportional hazards regression was used to assess the association between pMRI and recurrence, adjusting for potential confounders, including systemic therapies (chemotherapy and hormone therapy) and risk classification. Adjusted hazard ratios (HRs) with 95% confidence intervals (CIs) were reported. All statistical analyses were conducted using R version 4.3.1, and two-sided p-values <0.05 were considered statistically significant.

## 3 Results

### 3.1 Patient baseline characteristics

A total of 613 patients met the inclusion criteria, of whom 177 (28.9%) underwent pMRI and 436 (71.1%) did not (Table 1). The mean age of the cohort was 53.8 ± 10.5 years, and the majority of tumors were small, node-negative, and hormone receptor–positive. High-risk patients accounted for 75.4% of the study population. Baseline clinicopathologic characteristics were largely comparable between the pMRI and non-pMRI groups, including age, tumor size, nodal status, receptor profiles, lymphovascular invasion, and risk classification. Use of systemic therapy was also similar between groups. Notable differences included a higher proportion of standard radiotherapy in the pMRI group (87.0% vs. 74.8%, p = 0.001) and a shorter follow-up duration (5.4 ± 1.5 vs. 6.6 ± 2.7 years, p < 0.001), reflecting selection patterns and the later adoption of routine pMRI.

**Table 1:** Baseline characteristics of study population stratified by pre-operative MRI status.

Characteristic	Overall (n = 613)	Non-MRI (n = 436)	MRI (n = 177)	p-value
Demographics				
BMI (kg/m^2^)	25.0 ± 4.1	25.0 ± 4.2	25.0 ± 3.9	0.992
Age (years)	53.8 ± 10.5	53.8 ± 10.8	53.7 ± 10.0	0.807
**Tumor Characteristics**				
Tumor size (mm)	17.1 ± 10.5	17.2 ± 10.7	16.8 ± 9.9	0.693
pN-stage				0.410
N0	497 (81.9)	351 (81.1)	146 (83.9)	
N1 + N2	103 (17.0)	78 (18.0)	25 (14.4)	
N3	7 (1.2)	4 (0.9)	3 (1.7)	
ER/PR positive	503 (82.1)	354 (81.2)	149 (84.2)	0.449
HER2 positive	303 (49.4)	214 (49.1)	89 (50.3)	0.857
Lymphovascular invasion (+)	207 (36.6)	150 (36.9)	57 (35.9)	0.900
Section margin (+)	10 (1.6)	8 (1.9)	2 (1.1)	0.775
**High risk group**	462 (75.4)	330 (75.7)	132 (74.6)	0.852
**Systemic Therapy**				
Hormone therapy	491 (80.1)	345 (79.1)	146 (82.5)	0.405
Target therapy	67 (10.9)	43 (9.9)	24 (13.6)	0.235
Chemotherapy				0.134
Neoadjuvant	51 (8.3)	31 (7.1)	20 (11.3)	
Adjuvant	335 (54.7)	247 (56.7)	88 (49.7)	
**Radiation Therapy**				0.001
WBRT	480 (78.3)	326 (74.8)	154 (87.0)	
IORT alone	133 (21.7)	110 (25.2)	23 (13.0)	
**Follow-up and Outcomes**				
Follow-up time (years)	4.5 ± 1.0	4.5 ± 1.0	4.6 ± 0.8	0.371
Locoregional recurrence	37 (6.0)	32 (7.3)	5 (2.8)	0.052
Distant metastasis	27 (4.4)	22 (5.1)	5 (2.8)	0.319
Overall recurrence	56 (9.1)	49 (11.2)	7 (4.0)	0.007
Death	33 (5.4)	24 (5.5)	9 (5.1)	0.991

Data were reported as mean ± SD or n (%). High-risk patients were defined as those not meeting all ASTRO 2016 “suitable” criteria, including: age ≥50 years, positive hormone status, tumor size ≤2 cm, node-negative disease, negative margins, and no lymphovascular invasion (LVI). BMI, body mass index; ER, estrogen receptor; PR, progesterone receptor; HER2, human epidermal growth factor receptor 2; WBRT, whole breast radiation therapy; IORT, intraoperative radiation therapy; SD, standard deviation.

### 3.2 Overall outcomes

In the entire cohort, overall recurrence was significantly lower in the pMRI group (4.0% [7/177] vs. 11.2% [49/436], p = 0.007). Locoregional recurrence showed a borderline reduction (2.8% [5/177] vs. 7.3% [32/436], p = 0.052), while distant metastasis rates were lower but not statistically significant (2.8% [5/177] vs. 5.1% [22/436], p = 0.319). Overall mortality was comparable between groups (5.1% [9/177] vs. 5.5% [24/436], p = 0.991, Table 1).

### 3.3 Outcomes in high-risk patients

Among high-risk patients (n = 462), overall recurrence remained significantly lower with pMRI (5.3% [7/132] vs. 14.6% [48/330], p = 0.009; Table 2). Locoregional recurrence showed a trend toward reduction (3.8% [5/132] vs. 9.4% [31/330], p = 0.066), whereas distant metastasis and overall mortality were similar between groups.

**Table 2:** Outcomes stratified by risk classification and pre-operative MRI status.

	High Risk		p-value	Low Risk		p-value
	Non-MRI	MRI		Non-MRI	MRI	
Number	330	132		106	45	
Locoregional recurrence	31 (9.4)	5 (3.8)	0.066	1 (0.9)	0 (0.0)	1.000
Distant metastasis	22 (6.7)	5 (3.8)	0.331	0 (0.0)	0 (0.0)	NA
Overall recurrence	48 (14.6)	7 (5.3)	0.009	1 (0.9)	0 (0.0)	1.000
Deaths	20 (6.1)	7 (5.3)	0.925	4 (3.8)	2 (4.4)	1.000

Data were reported as number (%). MRI, magnetic resonance imaging; NA, not applicable. High-risk patients were defined as those not meeting all ASTRO 2016 suitable criteria. Low-risk patients met all ASTRO suitable, including: age ≥50 years, positive hormone status, tumor size ≤2 cm, node-negative disease, negative margins, and no lymphovascular invasion LVI.

### 3.4 High-risk patients by clinical predictability

Among the 462 patients classified as ASTRO high-risk, the distribution based on clinical predictability revealed that the clinically suspicious high-risk group accounted for 86.6% (400/462) of the cohort, while the pathologically-driven high-risk group (LVI + despite other favorable clinical indicators) represented 13.4% (62/462).

The impact of pMRI on oncological outcomes was further analyzed across the two high-risk subsets. In the clinically suspicious high-risk Group (n = 400), pMRI was associated with a significant reduction in the overall recurrence rate, which decreased from 15.0% (43/286) in the non-pMRI group to 6.1% (7/114) in the pMRI group (p = 0.024). While the pathologically-driven high-risk group (n = 62) did not reach statistical significance (p = 0.328), a notable clinical trend was observed: none of the 18 patients (0.00%) who underwent pMRI experienced any recurrence, compared to an 11.36% (5/44) recurrence rate in those who did not receive pMRI. Consistently lower rates of locoregional recurrence and distant metastasis were observed in the MRI-screened patients across both subgroups, although these individual endpoints did not reach statistical significance (Table 3)

**Table 3:** Oncological outcomes in clinically suspicious high-risk patients according to pMRI use.

	Overall	Non-MRI	MRI	p-value
Number	400	286	114	
Locoregional recurrence	33 (8.3)	28 (9.8)	5 (4.4)	0.116
Distant metastasis	25 (6.3)	20 (7.0)	5 (4.4)	0.453
Overall recurrence	50 (12.5)	43 (15.0)	7 (6.1)	0.024
Deaths	23 (5.8)	17 (6.0)	6 (5.3)	0.973

### 3.5 High-risk patients by radiotherapy modality

In high-risk patients treated with non-standard RT, IORT alone, recurrence patterns were similar regardless of pMRI use. In contrast, among those receiving standard radiotherapy, the pMRI group demonstrated significantly lower overall recurrence (4.9% [6/122] vs. 13.1% [35/267], p = 0.024), while locoregional recurrence, distant metastasis, and mortality did not differ significantly (Table 4).

**Table 4:** Recurrence patterns and survival outcomes according to MRI use, risk stratification, and radiotherapy modality.

	High Risk - IORT		p-value	High Risk - WBRT		p-value	Low Risk - IORT		p-value	Low Risk - WBRT		p-value
	Non-MRI	MRI		Non-MRI	MRI		Non-MRI	MRI		Non-MRI	MRI	
Number	63	10		267	122		47	13		59	32	
Locoregional recurrence	13 (20.63)	1 (10.00)	0.718	18 (6.74)	4 (3.28)	0.256	1 (2.13)	0 (0.00)	1.000	0 (0.00)	0 (0.00)	NA
Distant metastasis	1 (1.59)	0 (0.00)	1.000	21 (7.87)	5 (4.10)	0.245	0 (0.00)	0 (0.00)	NA	0 (0.00)	0 (0.00)	NA
Overall recurrence	13 (20.63)	1 (10.00)	0.718	35 (13.11)	6 (4.92)	0.024	1 (2.13)	0 (0.00)	1	0 (0.00)	0 (0.00)	NA
Total deaths	3 (4.76)	2 (20.00)	0.272	17 (6.37)	5 (4.10)	0.508	0 (0.00)	0 (0.00)	NA	4 (6.78)	2 (6.25)	1

Data were reported as number (%). MRI, magnetic resonance imaging; NA, not applicable. High-risk patients were defined as those not meeting all ASTRO 2016 suitable criteria. Low-risk patients met all ASTRO suitable criteria including: age ≥50 years, positive hormone status, tumor size ≤2 cm, node-negative disease, negative margins, and no lymphovascular invasion LVI.

### 3.6 Low-risk patients by radiotherapy modality

Recurrence events were rare among low-risk patients. In the IORT subgroup, one recurrence occurred in the non-pMRI group (2.1%) and none in the pMRI group. In the WBRT subgroup, no recurrences occurred in either group. Locoregional and distant failures were extremely uncommon, and mortality was low and comparable between groups (Table 4).

### 3.7 Cox regression analysis

In univariate analyses, pMRI was associated with a lower risk of overall recurrence (HR 0.40, 95% CI 0.20–0.90, p = 0.026). Hormone therapy was also protective (HR 0.30, 95% CI 0.20–0.60, p < 0.001), while chemotherapy and targeted therapy were associated with higher recurrence. BMI and radiotherapy modality were not significant predictors. In multivariate analysis adjusting for systemic therapies and radiotherapy modality, pMRI remained independently associated with reduced recurrence (adjusted HR 0.40, 95% CI 0.20–0.90, p = 0.036), and hormone therapy remained protective (adjusted HR 0.50, 95% CI 0.30–0.80, p = 0.008). Estimates for the low-risk group were unstable due to the very small number of events and are not interpretable (Table 5).

**Table 5:** Univariate and multivariate Cox regression analysis of factors associated with overall recurrence.

	Crude HR (95% CI)	p-value	Adjusted HR (95% CI)	p-value
pMRI (vs. no pMRI)	0.4 (0.2 - 0.9)	0.026	0.4 (0.2 - 0.9)	0.036
Low risk group (vs. high risk)	9.4 × 10^−9^ (0.0 - Inf)	0.990	0.0 (0.0 - Inf)	0.995
BMI ≥24 kg/m^2^ (vs. <24)	1.1 (0.6 - 1.9)	0.870	1.2 (0.7 - 2.1)	0.592
Chemotherapy (vs. no chemotherapy)	2.0 (1.3 - 3.1)	0.003	1.3 (0.7 - 2.2)	0.408
Hormone therapy (vs. no hormone therapy)	0.3 (0.2 - 0.6)	<0.001	0.5 (0.3 - 0.8)	0.008
Target therapy (vs. no target therapy)	2.2 (1.1 - 4.4)	0.029	1.8 (0.9 - 3.8)	0.125
RT group: IORT alone (vs. WBRT)	0.8 (0.4 - 1.7)	0.560	1.3 (0.6 - 2.8)	0.511

pMRI, pre-operative magnetic resonance imaging; HR, hazard ratio; CI, confidence interval; BMI, body mass index; RT, radiation therapy; IORT, intraoperative radiation therapy; WBRT, whole breast radiation therapy. Note: HR for low-risk vs. high-risk group could not be estimated due to very few events in the low-risk group.

## 4 Discussion

### 4.1 Risk-stratified utility of pMRI: the ASTRO reference framework

The principal finding of this study is that the oncologic benefit of pMRI in BCS patients is not universal but highly dependent on the patient's risk profile, establishing the ASTRO risk classification as a robust reference framework for guiding pMRI selection [18,19]. While the ASTRO criteria were updated in 2024 (Shaitelman et al.), our study intentionally maintained the 2016 risk stratification as the foundational framework for our institutional electronic kV-IORT protocol. Although ASTRO criteria were originally designed to determine the intensity of RT for residual breast tissue, our findings demonstrate that they also effectively identify patients at a significantly elevated risk of harboring occult disease [5,20]. While routine pMRI remains controversial, previous studies may have masked its subgroup-specific benefits by failing to stratify patients by risk and including total mastectomy cases driven by false positives, which diluted the observable oncologic impact within breast-conserving cohorts. By focusing specifically on the risk of residual disease in BCS patients, this stratified approach allowed us to identify significant oncological differences, establishing pMRI as a crucial safeguard when applied to the appropriate risk categories. By utilizing this framework, clinicians can move beyond the general controversy and identify which patients truly derive a survival benefit from advanced pre-operative imaging [9].

### 4.2 The decision-making role of pMRI within the ASTRO framework

Our results demonstrate that the clinical utility of pMRI is most pronounced when guided by the ASTRO risk framework. In the clinically suspicious high-risk group, where risk factors such as young age, tumor size, or suspicious nodal status were identifiable preoperatively, the implementation of pMRI led to a significant 9% absolute reduction in recurrence risk (15.0% to 6.1%, p = 0.024). This finding underscores the value of pMRI as a robust imaging reference framework for patients whose high-risk profile is already clinically evident, ensuring that occult multifocality or disease extent is adequately managed before breast-conserving surgery.

Conversely, for the Pathologically-Driven High-Risk group, our study addresses the clinical challenge of managing risks that are not preoperatively identifiable, such as LVI. Although the inability to predict LVI represents an inherent limitation of any preoperative framework, our findings offer a balanced perspective on pMRI utilization in this subset.

On one hand, the lack of statistical significance (p = 0.328) suggests that the omission of pMRI based on favorable preoperative indicators does not necessarily compromise standard oncological outcomes, as dictated by current clinical predictability. On the other hand, the absolute absence of recurrence (0%) among patients who underwent pMRI—compared to 11.4% in those who did not—indicates that the inclusion of pMRI is not a misplaced resource; rather, it provides a valuable protective trend by capturing occult disease. Therefore, while pMRI may not be mandatory for this specific group due to the lack of statistical evidence, its selective use remains a clinically sound strategy for ensuring optimal local control in the face of biological uncertainty.

In summary, the ASTRO framework provides a reliable baseline for pMRI decision-making, while the presence of ‘Pathologically-Driven’ cases highlights a category where pMRI offers supplemental clinical security rather than a mandatory requirement.

### 4.3 Synergistic benefits in high-risk patients: optimization, not substitution

Our analysis shows that pMRI significantly reduces recurrence in high-risk (ASTRO non-suitable/cautionary) patients by nearly two-thirds (5.3% vs. 14.6%, p = 0.009). This benefit is primarily driven by improved locoregional control (3.8% vs. 9.4%, p = 0.066), confirming pMRI's role in detecting occult lesions and achieving a “cleaner” surgical bed in patients most susceptible to subclinical disease burden [4,5].

Critically, the adjusted Cox regression validates pMRI as an independent protective factor (HR 0.42, 95% CI: 0.19-0.93, p = 0.036), but only when integrated with standard RT. In high-risk patients receiving standard RT, pMRI reduced recurrence by more than half (4.9% vs. 13.1%, p = 0.024); however, this benefit vanished in patients receiving non-standard inadequate RT (p = 0.718).

This interaction provides a crucial clinical insight: pMRI-guided surgical optimization cannot compensate for inadequate radiation coverage in high-risk patients. Instead, pMRI and standard RT work in synergy [6,21]. The imaging serves to minimize the macroscopic and subclinical disease burden, creating the ideal “low-burden” environment for standard radiation to effectively sterilize any remaining microscopic disease [22,23]. Failure to provide standard RT in this high-risk cohort, even after a pMRI-guided “clean” resection, still leaves the patient at a high risk of failure [24]. Thus, pMRI should be viewed as a tool to ensure the success of comprehensive treatment, not as a justification for treatment de-escalation in high-risk individuals.

### 4.4 pMRI in low-risk patients: from routine utility to a gatekeeper for de-escalation

In sharp contrast to the high-risk group, pMRI provided no discernible oncologic benefit for low-risk (ASTRO suitable) patients in our study. In this cohort, recurrence rates remained universally low regardless of pMRI utilization, reinforcing the premise that routine pre-operative imaging can be safely omitted in patients with minimal disease burden to avoid over-diagnosis and unnecessary surgical delays [9,21].

However, the clinical utility of pMRI in this low-risk population is currently undergoing a paradigm shift. Emerging data from the prospective PROSPECT trial (2024) suggest that pMRI findings in clinically low-risk patients might serve as a crucial clinical clue for safely omitting adjuvant RT altogether [25]. Central to the PROSPECT framework is not only the absence of MRI-detected occult lesions but also the requirement for low background parenchymal enhancement (BPE), as high BPE can obscure small malignancies and reduce the negative predictive value of the scan. By identifying a subset of patients with ‘clean’ MRI findings and minimal parenchymal interference, clinicians can isolate a population where the risk of recurrence is so low that RT provides no additional benefit. This distinction is key: while pMRI is non-essential for routine screening in low-risk patients, it becomes an essential imaging confirmation and biological gating step when considering RT de-escalation strategies, such as APBI [4,26]. In the low-risk setting, pMRI's primary value thus shifts from optimizing treatment coverage to ensuring the biological safety of de-escalation.

### 4.5 Limitations

Several limitations warrant consideration. First, the retrospective design introduces potential selection bias, as evidenced by baseline differences in RT type between pMRI and non-pMRI groups. While we controlled for these factors through stratification and multivariable analysis, unmeasured confounders may remain. Second, the shorter follow-up in the pMRI group could underestimate late recurrences, though our Cox regression analysis adjusted for this factor. Third, breast MRI interpretation is inherently operator-dependent. While our study benefited from high internal consistency by having all images evaluated by a single, highly experienced breast radiologist, this approach may limit the generalizability of the findings. The diagnostic accuracy of pMRI could vary in clinical settings with different levels of imaging expertise or institutional protocols [9]. Additionally, as this was a single-center study, institutional differences in patient selection, imaging interpretation, and treatment strategies may limit the generalizability of the findings. Fourth, the non-standard RT group (IORT alone in high-risk group) represents earlier practice patterns and may not reflect contemporary approaches. Finally, our sample size limited power for some subgroup analyses, particularly in low-risk patients.

### 4.6 Future directions

Prospective randomized trials specifically enrolling high-risk BCS patients are needed to confirm our findings. Such studies should mandate standardized RT and incorporate modern molecular biomarkers and the updated **2024 ASTRO guidelines** to further refine risk stratification [20] Cost-effectiveness analyses comparing risk-stratified versus universal pMRI strategies would inform policy decisions. Additionally, investigating whether specific MRI findings (e.g., extent of additional disease detected) predict which high-risk patients derive maximal benefit could enable even more precise imaging allocation. This study exclusively analyzed patients who successfully underwent BSC. Therefore, the rate of occult disease detection by pMRI and its subsequent impact on surgical conversion (e.g. from BSC to mastectomy) was not assessed. While this falls outside the scope of our current focus on long-term oncologic outcomes, it remains a compelling area for future investigation.

### 4.7 Conclusions

Ultimately, our findings refine current practice by providing a clear framework: the ASTRO risk classification should serve as the primary reference framework for pMRI utilization. By prioritizing pMRI for high-risk patients where the synergy with RT is most potent, while utilizing it selectively to ensure the safety of treatment de-escalation in low-risk patients, clinicians can maintain optimal oncologic outcomes while improving resource allocation and reducing the global healthcare burden.

## Funding

This study did not receive any specific grant from funding agencies in the public, commercial or not for profit sectors.

## CRediT authorship contribution statement

**Hsin-Yi Yang:** Conceptualization, Data curation, Formal analysis, Writing – original draft, Writing – review & editing. **Yi-Li Lin:** Conceptualization, Data curation, Writing – original draft, Writing – review & editing. **Cheng-Yen Lee:** Writing – review & editing. **Li-Chung Yang:** Conceptualization, Writing – review & editing. **Yu-Chen Hsu:** Conceptualization, Supervision, Validation, Writing – review & editing.

## Declaration of competing interest

The authors declare that they have no known competing financial interests or personal relationships that could have appeared to influence the work reported in this paper.
